# Disparities in Antenatal Care Visits between Urban and Rural Ethiopian Women

**DOI:** 10.1155/2023/9031344

**Published:** 2023-09-27

**Authors:** Senahara Korsa Wake, Abera Botore, Ahmed Mohammed, Kolato Gemede, Moyata Bariso, Urge Gerema

**Affiliations:** ^1^College of Natural and Computational Sciences, Ambo University, Ambo, Ethiopia; ^2^Department of Health Policy and Management, Jimma University, Jimma, Ethiopia; ^3^Department of Health Communication and Health Behavior, Jimma University, Jimma, Ethiopia; ^4^Department of Nutrition and Dietetics, Jimma University, Jimma, Ethiopia

## Abstract

**Background:**

Utilizing antenatal care is one of the best ways to identify issues that are already present or could arise throughout pregnancy. Despite increased efforts to expand health services and antenatal care utilization, less is known regarding antenatal care disparities across different population segments. Therefore, the purpose of this study was to assess the degree of discrepancies between urban and rural Ethiopian pregnant women's use of antenatal care.

**Methods:**

A total sample of 3927 women who gave birth to living children between 2014 and 2019 was included in the study from the 2019 Ethiopia Mini Demographic and Health Survey. Negative binomial Poisson's regression was adopted to analyze the data.

**Results:**

The majority of pregnant women (73.8%) attend at least one antenatal care. Pregnant women in rural areas visited fewer number of antenatal care (68.36%) than those in urban areas (90.1%). Women with age range of 30-40 (IRR: 4.56, 95% CI: 1.07-19.34), women with attending incomplete primary education (IRR: 0.05, 95% CI: 0.02-0.12), women with attending complete primary education (IRR: 0.17, 95% CI: 0.07-0.42), women from middle-income households (IRR: 0.12, 95% CI: 0.06-0.24), women from richer household (IRR: 0.26, 95% CI: 0.14,0.5), women from the richest household (IRR: 0.45, 95% CI: 0.24-0.86), and pregnant women from rural areas (IRR: 0.615, 95%: 0.56-0.67) were observed to be linked with the frequency of antenatal care visits.

**Conclusion:**

In Ethiopia, three-fourths of pregnant women attend at least one antenatal care. Place of residence, educational attainment, age in five years' group, and wealth index for urban/rural were related to the frequency of antenatal care visits.

## 1. Introduction

According to the World Health Organization (WHO), “every pregnant woman and newborn receives quality care throughout the pregnancy, childbirth, and postnatal period” [1]. Utilizing antenatal care (ANC) is one of the best methods for spotting issues that are already present or could arise during pregnancy [2]. Preparation for birth, evaluation of the mother and fetus, information on pregnancy risk indicators, nutritional counseling, and detection and management of obstetric difficulties are all included in ANC [3]. However, achieving a high coverage of high-quality prenatal care, skilled care at birth, and postnatal care for mother and baby could avert half of under-five fatalities, which happen among newborn newborns [4]. The high maternal mortality rate in many parts of the world is a result of unequal access to high-quality medical care. In 2015, problems from pregnancy and childbirth killed almost 303000 women and adolescent girls [5].

According to the WHO, ANC is the care given to pregnant women and teenage girls by trained medical professionals to ensure the best conditions for mother and child during pregnancy [6]. A woman who attends at least 8 ANC visits has a 61-time lower risk of dying from pregnancy-related causes than one who attends no ANC visits [7]. A study on the use of ANC services in Ethiopia revealed regional variations in the outcomes. The percentage of ANC services used ranged from 25% to 96% in the Somali region and Addis Ababa, respectively [8].

Even though expanded efforts to promote access to healthcare and ANC usage are crucial for further improving mother and child health, less is known regarding discrepancies in ANC among different segments of the population, and factors impacting the use of these services should be recognized [9–11]. Therefore, this study used data from the 2019 Ethiopia Mini Demographic and Health Survey to explore the scope of urban-rural discrepancies in the use of ANC services and factors that can be linked to the observed variations.

## 2. Materials and Methods

### 2.1. Study Area and Data Source

The study was done in Ethiopia. Ethiopia is home to various ethnic groups and cultural diversity, with its population speaking more than 80 different languages. There are two city administrations and nine regional states in Ethiopia [9, 12]. The 2019 EDHS, which is publicly accessible at http://www.dhsprogram.com/, served as the source of the data.

### 2.2. Study Population, Sample Size, and Sampling Procedure

The sample for the 2019 EDHS was made to offer estimates of important indicators for the nation overall, for urban and rural areas separately, and for each of the nine regions and the two administrative cities. The sample was selected in two stages.

In the first stage, 305 enumeration areas (93 in urban areas and 212 in rural areas) were chosen using a probability proportional method. From January to April 2019, a household listing operation was conducted in each of the designated enumeration zones. In the second round of selection, a specified number of 30 households per cluster were chosen from the newly produced household listing with an equal likelihood of systematic selection. All females between the ages of 15 and 49 were eligible to participate in the survey [9].

### 2.3. Study Variables and Data Analysis

The number of ANC visits each woman had during her most recent pregnancy served as the response variable. Residence, educational level, educational standing of the husband, age of the women, autonomy of women for health care, wealth index for rural/urban, age in 5-year groups, birth order number, and area were independent factors included in the study.

#### 2.3.1. Wealth Index

We gave scores to households according to the number and types of consumer goods they own, ranging from a television to a bicycle or car, and housing features, including flooring materials, toilet facilities, and source of drinking water. National wealth quintiles are compiled by assigning the household score to each usual household member, ranking each person in the household community by individual score, and after separating the distribution into five equal categories (poorest, poorer, middle, richer, and richest), each comprising 20% of the population [13].

### 2.4. Data Processing and Analysis

Information was gathered from all qualified women aged 15 to 49. Inquiries about these women's backgrounds, reproductive processes, use of contraception, pregnancies, and postpartum care were the main subjects of the questions.

The 2019 EMDHS interviewers recorded the interviewees' responses using tablets. The computer-assisted personal interviewing system's remote electronic file transfers, including the transmission of assignments from supervisors to interviewers and completed questionnaires from interviewers to supervisors, were made possible by the tablets' Bluetooth technology. The electronic data collection system deployed in the 2019 EMDHS was developed by the DHS Program using the mobile version of the Census and Survey Processing (CSPro) System [10]. First, a statistical test was performed on SPSS version 25 using Poisson's regression to determine whether overdispersion exists. We detected a potential problem with overdispersion with a scale factor (value/DF) greater than 1. Then, we used negative binomial Poisson's regression for the analysis, which can handle the problem.

## 3. Results

### 3.1. Sociodemographic Characteristics of Participants

The current study showed that overall three-fourths (73.8%) of pregnant women visit at least one antenatal care. Pregnant women in rural areas had 68.36% fewer antenatal care visits on average than those in urban areas (90.1%). As we can see from Table 1, about 74.8% of the women were from rural Ethiopia. Only 25% of pregnant women in rural Ethiopia received at least four ANC visits during their pregnancies, compared to 25% of women living in urban areas, where 9% of pregnant women received at least four ANC visits. The mean age of women who gave birth in five years was 34.62 (standard deviation of ±7.63) years. Approximately 30.8% of women were within the age range of 25–29 years. Over 51.9% of the women had no education, while 28.1% had incomplete primary education. With regard to wealth, 23.6% of the women fell in the wealthiest quintile, and 28.1% were grouped in the poorest quintile.

Urban residence areas had a larger mean number of ANC visits (5.32) than rural residence areas (2.62). The mean number of ANC visits varied significantly by age group, with women in the 30 to 34 age group having the highest number of visits (3.47), while women in the 45 to 49 age group had the lowest mean number of visits (1.94). Women with a complete secondary education had the highest mean number of ANC visits (7.73) compared to women with no education (2.14). Women who lived in households with the highest household wealth index had the highest mean numbers of ANC visits (4.74) (Table 1).

### 3.2. Factors Related to ANC Visits during Pregnancy

To investigate factors related to ANC visits, negative binomial regression was used. Type of residence, educational attainment, age in five years' group, and wealth index for urban/rural were related to the ANC visits during pregnancy at *p* value < 0.05. Women between the ages of 30 and 34 had 7% more prenatal care visits than women between the ages of 15 and 19 (IRR: 4.56, 95% CI: 1.07-19.34). Compared to women who had no education status, women who attended incomplete primary education and complete primary education had higher antenatal care visits (IRR: 0.05, 95% CI: 0.02-0.12; IRR: 0.17, 95% CI: 0.07-0.42), respectively. On the other hand, women from household's middle-income, richer household, and richest household had more antenatal care visits compared to women from poorer families (IRR: 0.12, 95% CI: 0.06-0.24; IRR: 0.26 (0.14, 0.5); IRR: 0.45, 95% CI: 0.24-0.86), respectively. Furthermore, pregnant women from urban areas visited antenatal care more frequently than women from rural areas (IRR: 0.615, 95% 0.56-0.67) (Table 2).

## 4. Discussion

The current study found that a significant portion of the participants (three-fourths of the women) were from rural Ethiopia, and only one-third of pregnant women in these areas had at least four ANC visits during their pregnancies. When compared to earlier Ethiopian demographic health surveys conducted in 2000, 2005, 2011, and 2011, which found that 27.6%, 28.2%, 34.5%, and 62.9% of women lived in urban areas, respectively, nearly half of the pregnant women had at least four ANC visits. This indicates an increase in ANC utilization [14]. This improvement may be due to increased awareness creation activities, health promotion, health coverage, and skilled health professional increment in rural area. There were still disparities from region to region and from rural to urban regarding antenatal care visits. This might result from inequalities in accessibility of maternal and child health, the disparity in the number/skill and commitment of health professionals of access to education, absence/poor transportation, and country policy and program implementation differences between rural and urban regarding maternal health services.

In this study, place of residence, educational attainment, age in five years' group, and wealth index for urban/rural were associated with the number of ANC visits during pregnancy. The present study showed that women aged groups 30 to 34 and 40 to 44 years had seven and three percent more ANC visits compared to women aged 15-19 years, respectively. This finding was supported by studies done in different countries [14–16] that showed the association between increased age and antenatal care visits of the women. Birth complications and health conditions of the women are higher in older women which increase the demand for antenatal care visit. Additionally, the current study found that women who attended school had more antenatal care visits than women who did not attend school. This study is in line with the study done in Ghana [17] and Congo [18].

Furthermore, the results of negative binomial regression showed that the wealth index of the household had a significant effect on a number of ANC visits during pregnancy. The pregnant women from middle income had twelve percent more likely to visited ANC when compared to pregnant women from poor households. Similarly, pregnant women from richer households and richest households had fourteen and twenty-four percent more antenatal care visits when compared to women from poor households. The finding is similar to studies conducted in different countries [19, 20]. The current study also showed that, compared to women from rural areas, women from urban areas had 61 percent more antenatal care visits.

### 4.1. Limitation of the Study

The nature of the data from EDHS is cross-sectional type in which the temporal relationship between the outcome variable and predictors could not be assessed; EDHS data is a questionnaire-based survey that could recall biased and incomplete data. The use of a large sample size and a wide geographic scope are some of the strengths of the study.

## 5. Conclusion

The current study showed that overall three-fourths (73.8%) of pregnant women visit at least one ANC. Pregnant women in rural Ethiopia experienced fewer ANC visits on average than those in urban. Type of residence, educational attainment, age in five years' group, and wealth index for urban/rural were related to the frequency of ANC visits during pregnancy.

## Figures and Tables

**Table 1:** Sociodemographic characteristics and the number of pregnant women who visit ANC services.

Variable	Category	*n*	%	Mean (95% CI)
Place of residence	Urban	1004	25.2	5.32 (4.67,5.97)
	Rural	2975	74.8	2.62 (2.46,2.78)

Age	15-19	246	6.2	2.79 (1.99,3.6)
	20-24	808	20.3	3.31 (2.89,3.74)
	25-29	1225	30.8	3.46 (3.1,3.82)
	30-34	809	20.3	3.38 (2.95,3.81)
	35-39	558	14	3.47 (2.77,4.17)
	40-44	247	6.2	2.85 (2.03,3.66)
	45-49	86	2.2	1.94 (1.5,2.39)

Educational attainment	No education	2065	51.9	2.41 (2.18,2.63)
	Incomplete primary	1119	28.1	3.67 (3.24,4.09)
	Complete primary	187	4.7	4.77 (3.32,6.22)
	Incomplete secondary	336	8.4	4.78 (3.97,5.5)
	Complete secondary	40	1	7.73 (3,12.45)
	Higher	232	5.8	5.42(4.58,6.26)

Wealth index	Poorest	1119	28.1	1.96 (1.6,2.31)
	Poorer	606	15.2	2.66 (2.31,3.01)
	Middle	642	16.1	3.42 (2.89,3.94)
	Richer	673	16.9	3.97 (3.4,4.54)
	Richest	939	23.6	4.76 (4.3,5.23)

Religion	Orthodox	1264	31.7	4.14 (3.76, 4.52)
	Catholic	25	0.6	2.08 (1.47, 2.69)
	Protestant	780	19.6	2.84 (2.56, 3.12)
	Muslim	1862	46.8	2.99 (2.66, 3.33)
	Traditional	35	8.8	1.26 (0.59, 1.92)
	Other	13	0.3	1.77 (0.78,2.76)

**Table 2:** Results of negative binomial regression.

Variable	Category	IRR (95% CI)	*p* value
Age	15-19	1	
	20-24	2.34 (0.46, 11.8)	0.304
	25-29	3.95 (0.91, 17.15)	0.067
	30-34	4.56 (1.07, 19.34)	0.040^∗^
	35-39	4.21 (0.97,18.28)	0.055
	40-44	4.61(1.03, 20.66)	0.046^∗^
	45-49	2.47 (0.49, 12.51)	0.275

Educational attainment	No education	1	
	Incomplete primary	0.05 (0.02, 0.12)	<0.001^∗^
	Complete primary	0.17 (0.07, 0.42)	<0.001^∗^
	Incomplete secondary	0.52 (0.15,1.84)	0.312
	Complete secondary	0.53 (0.18,1.58)	0.254
	Higher	10.04(1.12,89.71)	0.039^∗^

Wealth index	Poorest	1	
	Poorer	0.06 (0.03,0.12)	<0.001^∗^
	Middle	0.12 (0.06, 0.24)	<0.001^∗^
	Richer	0.26 (0.14,0.5)	<0.001^∗^
	Richest	0.45 (0.24,0.86)	0.016^∗^

Place of residence	Rural	1	
	Urban	0.615 (0.56-0.67)	<0.001^∗^

^∗^Significance at 5%. IRR: incidence rate ratio.

## Data Availability

The data used in this study is publicly available at https://dhsprogram.com.
